# Designing Fit for Purpose Health and Social Services for Ageing Populations

**DOI:** 10.3390/ijerph14050457

**Published:** 2017-04-25

**Authors:** Jean Woo

**Affiliations:** CUHK Jockey Club Institute of Aging and Department of Medicine & Therapeutics, The Chinese University of Hong Kong, Hong Kong, China; jeanwoowong@cuhk.edu.hk; Tel.: +852-2632-3493

**Keywords:** chronic disease, disability, frailty, intrinsic capacity, integrated care

## Abstract

Population ageing is occurring in all countries, regardless of the level of economic development. While the rising burden of chronic diseases and disabilities as a consequence of this demographic transition is well recognized, the increasing prevalence of geriatric syndromes as a public health issue is not as well recognized. Recently the World Health Organization’s World Health and Ageing Report emphasized functional ability as an important outcome for aging populations, highlighting the concept of raising intrinsic capacity throughout the life course. The complementary perspective is the prevention of frailty, which has physical, cognitive, social and psychological dimensions. Therefore, services for older people should encompass medical as well as social components. The need and evolution for a transition in health and social services in Hong Kong, a special administrative region of China which has a population with the world’s highest life expectancy, is presented as an example of how one developed economy attempts to meet the challenges of population ageing. There is a need to shift to integrated care in the community instead of specialty dominated hospital care, and to establish regular activities in the community to adopt and maintain a lifestyle that reduces frailty and disability (or promotes intrinsic capacity). A top down approach with financial incentives, together with public education to help drive policy changes, are key drivers of change. It is expected that there will be much heterogeneity between different countries in terms of barriers and facilitators, such that each country needs to document their needs and design appropriate services.

## 1. Introduction

With advances in public health measures, life expectancy in all countries at different stages of economic development is increasing because of reduced infant and maternal mortality rates, as well as a reduction in mortality from infectious diseases. This demographic transition is accompanied by an increase in chronic disease prevalence and disability rates, as shown in the Global Burden of Disease reports. This series documents the prevalence of various chronic diseases and estimates disability-free life expectancy for various countries [1,2]. The World Health Organization (WHO) adopted a different approach to promote Ageing and Health as a lifelong goal to pursue. As a public health response to ageing, WHO places emphasis on promoting or maintaining the intrinsic capacity of people as they age, not only through modifying individual lifestyle behavior, but also by promoting enabling physical or social environments that facilitate functional capacity even in the presence of chronic diseases and disabilities [3,4]. Furthermore, the WHO outlined a public health framework for action to foster this model for Healthy Ageing in a draft document tabled at the 69th World Health Assembly on 22 April 2016, which was widely supported.

**Figure 1:**
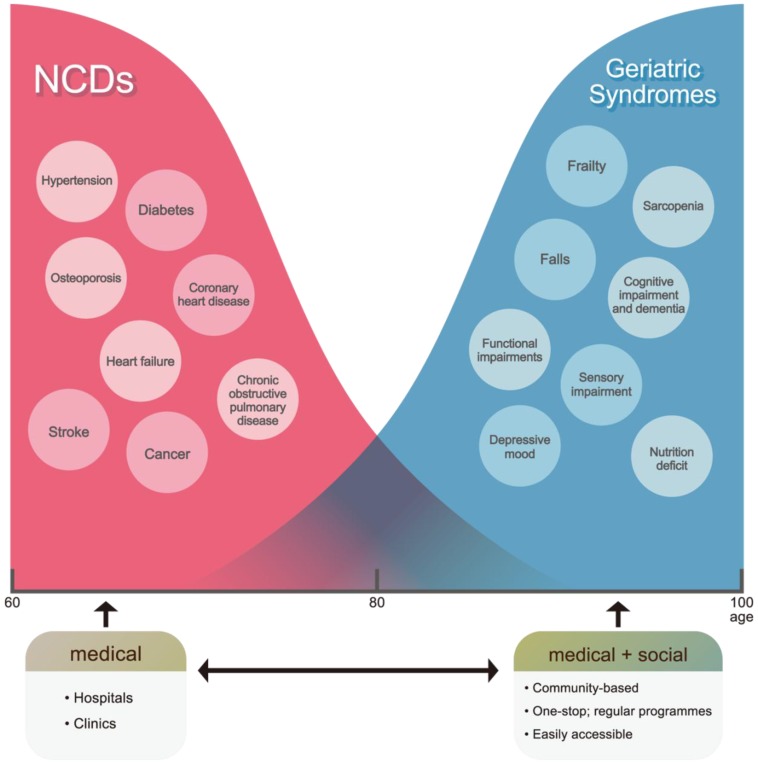
Non-communicable diseases (NCD) and frailty.

The opposite of functional capacity may be considered frailty, representing the loss of homeostasis or physiological reserves with ageing and associated with many adverse health outcomes [5]. Frailty represents one of the geriatric syndromes which are commonly encountered with increasing age, such that management of these syndromes is becoming increasingly important (Figure 1). While there is a generally decreasing trend in incidence of common chronic diseases such as ischaemic heart disease and osteoporosis, there appears to be an increasing trend for increasing frailty in successive cohorts [6,7]. This indicates that increasing life expectancy worldwide may be accompanied by increasing levels of frailty, resulting in increased disability, and use of various health and social care resources. The syndrome is considered to include physical, cognitive, social, and psychological domains, and this relates to the concept that intrinsic capacity may be affected by these factors. The overall prevalence of frailty and pre-frailty in the community-dwelling population has been reported to be approximately 10% and 50% respectively using different definitions [8,9]. Older people living with frailty are at risk of marked changes in physical and mental health after minor events which challenge their health, commonly leading to falls, disability, hospitalization, institutionalization, and mortality. There is a concomitant increase in caregiving burden and healthcare expenditures. As a result, frailty is beginning to guide the development of health policies in caring for older people [10,11,12,13].

Historically, the goal of public health was to reduce mortality from infectious diseases, and then to reduce mortality from chronic diseases. Today, public health goals also include physical function, psychological wellbeing [4,14,15], and prevention of geriatric syndromes such as frailty. Furthermore, the WHO draft global strategy calls for multisectoral action for a life course approach to healthy ageing, with a plan of action endorsed by the World Health Assembly for all governments to pursue. The importance of healthy ageing was emphasized as a public health priority, with the implementation to be based on a sustainable and evidence based policy response. This strategy complements existing initiatives relating to ageing populations such as age-friendly cities and communities, developing person-centred and integrated care, tackling dementia and providing palliative care. The public health framework for healthy ageing promoted by the WHO calls for four key areas of action by governments on healthy ageing [4]: aligning health systems to the needs of the older populations they now serve; developing systems to provide long term care; ensuring everyone can grow old in an age-friendly environment; and improving measurement, monitoring, and understanding.

**Figure 2:**
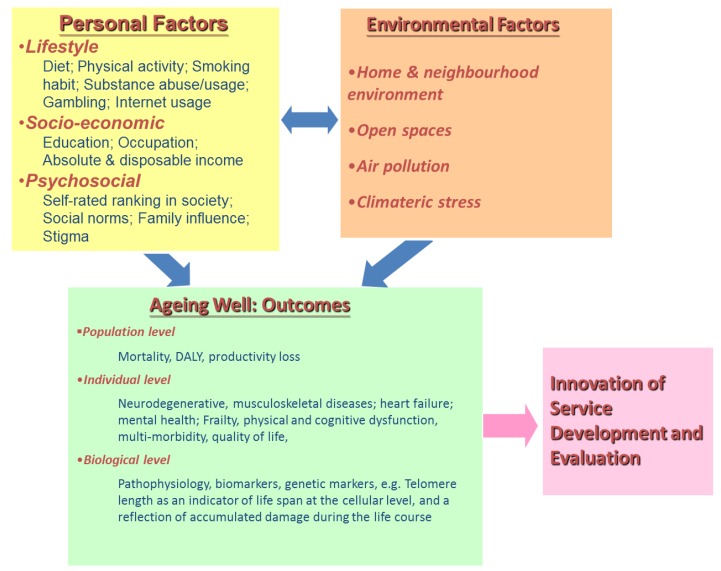
An integrated framework.

In order to implement any of the above actions, information relating to the epidemiology of factors contributing to healthy ageing is required, the availability of which varies between different countries with different socioeconomic, lifestyle and physical and social environments. A holistic framework is needed to examine all contributory factors, and ideally the relative magnitude of the contributing individual risk factors (Figure 2). Consequently, there is a need to integrate medical and social services for older people. It is possible that higher income countries with highly developed services for separate organ specialties may face more problems as more redesign and inter organizational barriers need to be overcome. For countries with rudimentary services for older people, a new structure may be developed based on available resources using a step care model using simple community screening followed by a care algorithm.

The adoption of integrated models of care for ageing populations is dominating aging health policy discourse in many countries, where the focus shifts from acute care to prevention and maintenance of chronic conditions and where primary health and social care are central to delivering quality integrated services. Key features of integrated care include person-centredness, empowerment of the care recipient, multidisciplinary teams with efficient communication, and cooperation between formal and informal caregivers. With this background, we used Hong Kong, a Special Administrative Region of China, as a case study to illustrate how health and social services should be redesigned to meet the needs of the ageing population, following the WHO healthy aging framework and strategy for action. Hong Kong is particularly suitable in that it now has the highest life expectancy in the world with those 65 years and older constituting 15% of the population in 2015.

## 2. Hong Kong as a Case Study

Hong Kong has a comprehensive range of medical and social services, partly modeled after the UK. Primary care is predominantly provided by the private sector; hospital services are largely provided by the government at very low cost. These services tend to be crowded with long waiting lists for specialist appointments and nonemergency investigations and operations, with very limited consultation time per patient. Various forms of government social welfare programs provide financial assistance. Community-based services are also available, consisting of day care centers and home-based care teams (social and health-care support). The current government policy is to promote aging in place in the community and reduce the demand for residential care homes for the elderly. The lack of easily accessible publicly funded primary care in Hong Kong has contributed to elderly services being shifted to the hospital system and has also contributed to a high rate of long-term institutional care, in addition to higher rates of avoidable mortality compared with other countries.

### 2.1. Documenting the Needs of the Ageing Hong Kong Population

Epidemiological data showed that while the incidence of various chronic diseases may be on a downward trend, the trend for mortality caused by these diseases follows a much steeper downhill trajectory. Since the absolute number of older people is increasing, the burden of chronic diseases, in particular dementia, is increasing [1,2]. The prevalence of pre-frailty and frailty among community-dwelling people aged 60 years and over is approximately 50% and 10% respectively [9]. Not surprisingly, this is accompanied by an increasing trend of dependency [6], and frailty [16] from age period cohort analyses. There is also a suggestion of ageism in service prioritization [17]. There is a high prevalence of depressive symptoms among older people aged 70 years and over (30% in men and 40% in women), while suicide rates are among the highest in the older age groups [18,19]. Psychological well-being among older people in Hong Kong ranked 79 out of 97 countries in the Global Age Watch Index in 2015, with respondents scoring poorly in eudaemonic wellbeing [20]. Loneliness, a psychological state that predisposes people to various chronic diseases is also commonly encountered among older people [21,22,23,24].

### 2.2. Designing Fit for Purpose Models of Care

According to the WHO global strategic objective 3, health systems should be aligned to the needs of older populations. In the case of Hong Kong, the health system is not yet aligned, in that the service is designed to react to specific diseases independently, to cure acute conditions, with fragmentation and lack of coordination. Problems commonly encountered in older people, such as chewing, visual and hearing difficulties, self-care, ambulation, instrumental activities of daily living, are seldom elicited or managed in the primary or secondary care setting. This is partly a result of failure in training curricula for healthcare professionals to incorporate principles of care of older persons. A health care system should be responsive to needs of older people with varying functional capacity from normal to declining to dependency, offering affordable access to integrated services.

In the past two years, awareness has increased on the concept of frailty that encompasses these multiple needs, as well as the need for prevention, early detection through community screening, and continuing support. A healthcare system that includes all of these elements is being planned and pilot models are being discussed. A report by the think tank Our Hong Kong Foundation proposed the use of a healthcare voucher system for management of chronic diseases, and the creation of health-enabling networks from current fragmented health and social care centers [25]. It also highlighted the need for empowerment and self-management, an age-friendly environment, as well as the use of technology in facilitating these goals.

With regard to the use of technology, an initiative to provide medical support to older people living in residential care homes for the elderly was pioneered about fifteen years ago. This involved the use of consultations through teleconferencing, using Polycom teleconference equipment and onsite television. Services included medical, psychiatric, nursing, dermatology, podiatry, and allied health consultations [26].

An experimental model of community care integrating medical and social components may address some of the current gaps in service needs for the older population in Hong Kong. In response to the needs identified in surveys and focus group studies, such a center was established eight years ago (Jockey Club CADENZA Hub: www.jcch.org.hk), as part of the Hong Kong Jockey Club CADENZA Project, an initiative to promote an elder-friendly Hong Kong (www.cadenza.org.hk). The objectives are to support frail elderly people with multiple morbidities in physical and psychological domains in the community using a case management approach, to support informal caregivers, to carry out health promotion and health maintenance (optimizing function, for those with chronic conditions), and to provide services designed from the user’s perspective taking into account gaps in current services. The program consists of three categories: health promotion and health maintenance programs for the soon-to-be old and independent elderly, an optimal lifestyle and disease control program for those with chronic conditions, and prevention of decline and regaining physical and or mental function in a day care setting. An important characteristic is that people would want to come to the center on a regular basis, as the environment is designed with a club atmosphere. Activities relating to health are incorporated, but not dominant. The service model is a self-sustaining, seamless, one-stop service that places the focus on raising health literacy and empowerment; it involves multiple partners and is transdisciplinary in nature. The underlying philosophy is that if services are designed to meet needs that are unsatisfied with existing services, people will be willing to pay. Such a center may also be used as a basis for future development of the step care approach for care of older people in the community, and could be particularly effective for mental health support. Use of services in this center is currently borne by the user, with the exception of day care which is supported by the government on a sliding scale according to a means test, such that the cost for day care may be entirely borne by the government. The cost may vary from HKD 2500 per month to nothing, depending on the means test. Other services vary from HKD 500 for individual consultation with allied health staff, optometry, to HKD 680 for frailty intervention groups twice a week for 12 sessions. Health insurance does not cover any of the care programs.

Since its inception, a large proportion of users of the day care section consist of older people with dementia, suggesting that there is a service gap for this group of people and their caregivers. Lifestyle modification programs with an emphasis on behavioral change were also popular, suggesting that this model may complement pharmacological treatment by doctors for many lifestyle-related diseases. Group exercise programs to prevent falls were devised as a combination of social and health-promoting activities in a social setting with regular sessions over a 36-week period, unlike the traditional physiotherapy sessions lasting for a shorter duration and without the social component. It was designed to ensure high compliance with the directed exercises. The self-reported factors that motivated elderly people to participate included noted beneficial effects on their activities of daily living with increased capacity to carry out housework and improved walking stability, improved regular and long-term scheduling, a manageable level of difficulty, and a comfortable and friendly environment with a group of peers [27]. Recently, programs for frailty intervention have also started; they have had an excellent response rate despite costing money and, in some cases, requiring long travel times to their locations.

In response to the WHO’s proactive approach to promoting intrinsic capacity through designing new systems for primary care for older persons living in the community, a step care approach in early detection of problems and a guide to subsequent action that allows for management in the community that reduces reliance on the hospital system, is being developed and piloted in Hong Kong. This model involves early detection of physical and cognitive frailty, as well as limitations in daily functioning and other unmet needs through community screening. For example, frailty may be detected by administering five questions (either self-administered or by volunteers) as part of a rapid geriatric assessment battery, as described in the report on community screening for frailty [9]. The latter should have wide coverage but not incur high cost, such as a step care approach. Furthermore, the model calls for empowerment and engagement of older people, thus creating an enabling environment and re-orienting the model of care. A recent survey of community dwelling people aged 60 years and older using a multi-domain screening questionnaire for older people identified 75% of people had memory problems, 64% had varying levels of frailty, 38% had chewing difficulties, 14% had sarcopenia, 14% had difficulties in instrumental activities of daily living, 12% had poor self-rated health, and 10% were not satisfied with life. All these problems are amenable to further assessments and action. However the current system does not respond very well to geriatric syndromes, but rather to specific diseases. Geriatric syndromes predispose older people to dependency, caregiver stress, and increase demands on health and social care services that are unable to provide quality care. An algorithm generating further questions and possible solutions can be constructed, with reference to appropriate services. Information technology can be developed to support this initiative, eventually linking users to seek help in community multidisciplinary health care centers led by doctors and nurses trained in care of older people, including in end of life care.

Currently the government is exploring various primary care models, such as setting up community health centers based on existing social service community centers under various non-government organizations where nurse-led prevention and health promotion programs as well as management of geriatric syndromes may be provided. These centers would be linked with family physicians and geriatricians in either public or private settings, or new centers could be established with one stop service that includes a primary care physician supported by allied health staff. These centers would provide continuing care in the community to complement the episodic care provided by hospitals (whether as inpatient or outpatient).

The above developments should be in the wider context of enabling environmental and psychosocial factors such as articulated by the WHO Age friendly City domains, as these are critical components to the WHO’s concept of preserving or raising intrinsic capacity, since the impact of social factors on health is well established [28].

## 3. Conclusions

To meet the challenges of ageing populations, the WHO has advocated healthy aging as a goal, with an emphasis on function or maintaining/promoting intrinsic capacity. This requires re-orientation of health policy and systems to shift to integrated care of older people in the community from specialty dominated hospital care, as well as regular activities in the community to adopt and maintain a lifestyle to reduce frailty and disability (or promote intrinsic capacity). Redesigning a responsive health system requires a top down approach with financial incentives to service providers, the development of information systems collecting data for intrinsic capacity (or frailty), as well as training for the health and social care workforce in care of older people. There will be heterogeneity between different countries in designing healthcare systems that meet the WHO healthy aging framework. Individual countries need to document their particular needs, as well as the barriers that exist and the facilitators needed to redesign the system.
